# Atomic-scale diffractive imaging of sub-cycle electron dynamics in condensed matter

**DOI:** 10.1038/srep14581

**Published:** 2015-09-28

**Authors:** Vladislav S. Yakovlev, Mark I. Stockman, Ferenc Krausz, Peter Baum

**Affiliations:** 1 Center for Nano-Optics and Department of Physics and Astronomy, Georgia State University, Atlanta, GA 30303, USA; 2 Max-Planck-Institut für Quantenoptik, Hans-Kopfermann-Str. 1 85748 Garching, Germany; 3 Ludwig-Maximilians-Universität München, Fakultät für Physik, Am Coulombwall 1, 85748 Garching, Germany

## Abstract

For interaction of light with condensed-matter systems, we show with simulations that ultrafast electron and X-ray diffraction can provide a time-dependent record of charge-density maps with sub-cycle and atomic-scale resolutions. Using graphene as an example material, we predict that diffraction can reveal localised atomic-scale origins of optical and electronic phenomena. In particular, we point out nontrivial relations between microscopic electric current and density in undoped graphene.

Any light-matter interaction is, at its fundamental level, defined by field-driven motion of charges in their atomic-scale environment. Depending on a material’s structure, potential-energy landscape, correlation mechanisms and applied field strength, this motion may be highly complex, and it causes a rich variety of macroscopic optical phenomena, which are at the heart of current laser technologies^1^, quantum optics^2^,^3^, spectroscopy^4^, nonlinear microscopy^5^,^6^, frequency comb metrology^7^,^8^, optical information processing^9^,^10^, and light-driven electronics^11^,^12^,^13^.

In this paper, we take a *real-space/sub-cycle perspective* seeking the most direct way to comprehend the nature of optical and electronic phenomena at lightwave frequencies. We consider the following questions: Within a laser cycle, what fraction of the material’s charge density is driven into which direction resulting in which macroscopic response? Are there certain electron densities that are particularly efficient for specific effects? And what experimental approach can possibly reveal such three-dimensional motion of charges in real space? Sub-cell and sub-cycle resolution in space and time, if feasible, would arguably provide the most straightforward access to the basic microscopic effects underlying complex light-matter interaction.

## Concept for sub-cycle imaging in real space

The example system considered in this work is graphene, which is well known for its unusual electronic and optical properties^14^ and high potential for future high-speed electronics. Here, graphene is chosen due to its relatively simple structure and the large number of valence electrons per nucleus. The imaging concept is ultrafast pump-probe diffraction with electron or X-ray pulses of sub-laser-cycle duration, short enough to freeze out the relevant electronic and resultant atomic motion driven by light. Such pulses are achievable with free-electron lasers and, potentially, also with electron pulses, as we discuss below. A series of diffraction snapshots recorded under such conditions for different delays with respect to the driving field can provide real-time insight into rearrangement of electronic charge in condensed matter with spatial and temporal resolution. This idea was introduced earlier^15^ and modelled in detail for electronic superposition states in atoms and diatomic molecules^16^,^17^,^18^,^19^,^20^, as well as for polyatomic molecules on much slower time scales^21^. Seminal experiments with femtosecond X-rays^22^,^23^,^24^ revealed sensitivity of ultrafast diffraction to light-field-driven electron dynamics, albeit in a cycle-averaged experiment without sufficient temporal resolution. Our main goal here is to demonstrate the feasibility and importance of fully resolving electronic motion driven by the oscillating light fields in space and time. We also show how graphene’s complex relations between atomic- and macroscopic-scale phenomena may be revealed by ultrafast pump-probe diffraction.

**Figure 1:**
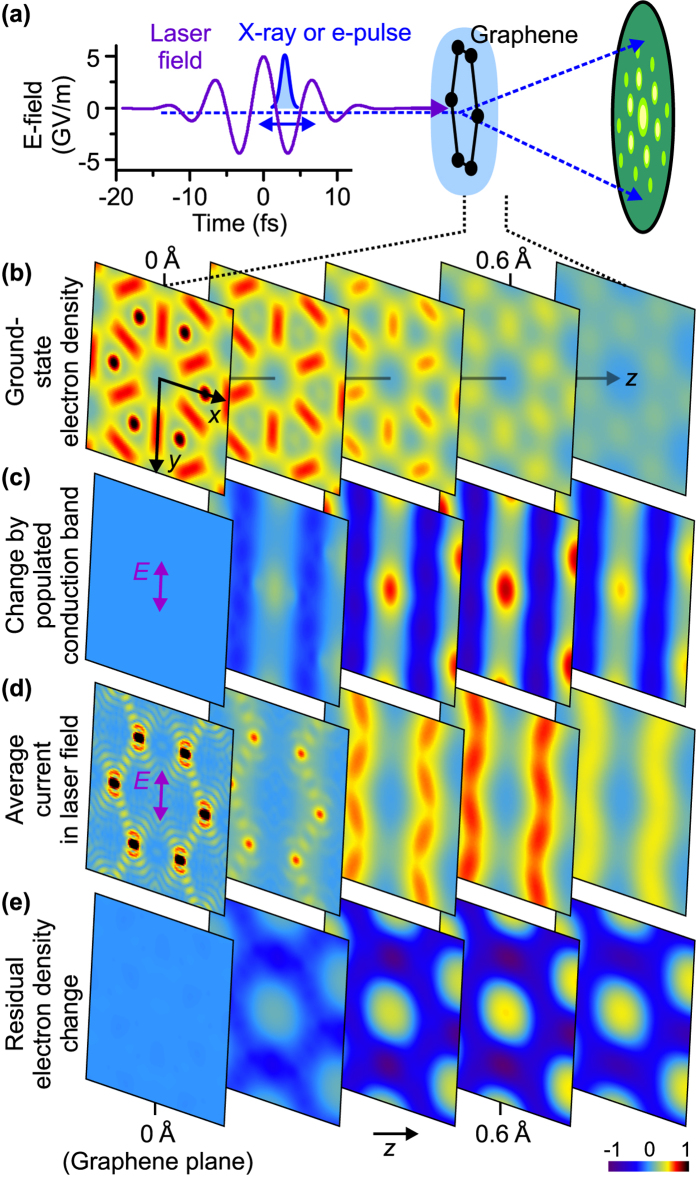
Real-space imaging of electronic motion in condensed matter. (**a**) A few-femtosecond electron or X-ray pulse (blue) probes how the charge density in a sample, here graphene, changes under influence of an intense mid-infrared laser pulse (violet). The intensities of Bragg spots (green) are measured for a series of delays between the pulses. (**b**) Ground-state electron density of graphene’s valence-band electrons in several planes parallel to the sample; the distance to the centre of the atomic layer varies from *z* = 0 in steps of Δ*z* = 0.2 Å. (**c**) Change of the electron density caused by excitation from the top 2 eV of the valence band below the Fermi energy to the bottom of the conduction band. (**d**) Time-averaged magnitude of the atomic-scale electric current. (**e**) Change of the microscopic electron density after the interaction with the laser pulse. In panels (**b**–**e**), the coordinates *x* and *y* span the range ±2.1 Å.

Figure 1(a) depicts a schematic of the proposed experiment. A material (here graphene) interacts with a 10-fs laser pulse carried at a frequency of 140 THz, i.e., a wavelength of 2.1 μm. For such a pulse, the direct excitation of phonons is negligible, and graphene’s response is mostly electronic. The 7-fs optical period of a 2.1-μm pulse is short enough to neglect, in a first approximation, lattice motion; at the same time, it allows for realistic sub-cycle diffraction measurements with few-fs electron and X-ray pulses. As a peak electric field, we use 0.5 V/Å, which is high enough for the onset of nonlinear and strong-field effects^25^ while still below graphene’s damage threshold^26^. Current laser technology readily provides such pulses^1^,^27^. The optical electric field in our calculations is polarised along graphene’s Γ-K direction, i.e. in real space from the hexagon centre towards the middle of a C-C bond. Snapshots of the moving charges are taken by pump-probe diffraction with ultrashort electron or X-ray pulses of sub-cycle duration for later slow-motion replay.

### Real-space electron dynamics and its complexity

First we report a simulation of graphene’s electron dynamics in real space and discuss its relation to macroscopically observable optical phenomena. The method employed is a numerical solution of the time-dependent Schrödinger equation for a phenomenological lattice potential; see Methods. Figure 1(b) shows the static density of graphene’s valence electrons without the optical field. From a chemical perspective, graphene is a sp^2^-hybridised structure; each carbon atom, which has electron configuration 1s^2^2s^2^2p^2^, contributes three of its four valence electrons to three sigma bonds and one to a delocalised electron density above and below the plane of the nuclei.

Hence, there are two electrons per sigma bond and two delocalised electrons per unit cell. In the computed ground-state charge-density map of Fig. 1(b), these are evident: the sigma bonds form density maxima between carbon atoms, and the *p*_*z*_ electrons contribute to an additional delocalised density at larger 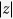. For sufficiently large 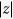, for example, at 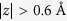, there are local density maxima above/below each C-C bond, not above/below the nuclei. This is a consequence of the overlapping *p*_*z*_ orbitals in conjunction with remaining parts of the bond-forming sp^2^ densities. In other words, the sigma bonds and the delocalised electron density overlap significantly in real space. We note that there is also some remaining density of the valence electrons concentrated around the nuclei.

Electric current in undoped graphene can only exist if some electrons are excited into conduction bands. Let us consider a gedankenexperiment where we transfer valence electrons within a 2-eV energy range from the Fermi level and below into the lowest conduction band conserving the quasi-momentum. Note that this includes the electrons occupying the vicinities of the K points in reciprocal space. Figure 1(c) depicts the change of the electron density upon such excitation. There is no change in the graphene plane, but a maximum change at a distance of 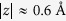 above and below it. Remarkably, the electron density is transferred from two localised stripes (dark blue) into a central area (yellow) with a peak (red) at the hexagon centre. This real-space representation of the static band-population change provides the first sign of electronic motion’s complexity in real space.

Next we discuss electron dynamics driven by the laser field. Generally, a material’s response can be separated into localised charge displacements and delocalised charge motion. At low, perturbative fields, the response of dielectrics for frequencies below the bandgap is due to localised charge displacement causing their polarisation. In contrast, electric current in conducting media (metals, doped semiconductors, conjugated polymers, etc.) is caused by delocalised charges. In general, especially in complex materials, both localised and delocalised charges shape the optical response.

In undoped graphene, microscopic (atomic-scale) electron and current densities are, to a significant degree, complementary to each other in real space. This is evident from Fig. 1(d) where we plot magnitude of current density averaged over time. The result looks dramatically different from both the ground-state electron density of Fig. 1(b) and also the excited-state density change of Fig. 1(c). Although the electron density transferred to the conduction band is concentrated in the unit cell centre [Fig. 1(c)], the current is concentrated at its periphery and 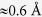 above and below the atomic plane where the delocalised and overlapping *p*_*z*_ orbitals are situated. Hence, the electric current predominantly flows through regions where these orbitals form continuous chains along the laser polarisation. Note that little or no current flows at the hexagon centre, where carbon orbitals have a negligible overlap. Also, there is no appreciable contribution to graphene’s local or non-local currents originating from any C-C bond electrons in the atomic plane. These combined observations further suggest value of a real-space visualisation approach.

Figure 1(e) identifies the spatial regions associated with optical absorption by depicting the residual change of electron density at *t* = 20 fs, after the excitation pulse has passed. The deposited energy per pulse is, in our model, 1.9 eV per unit cell. This causes no density change in the plane of the nuclei, but above and below it, charge is transferred towards the carbon hexagon centre where the initial electron density is low. This effect of the intense laser pulse is qualitatively different from that shown in Fig. 1(c) where electrons from the top of the valence band are artificially transferred to the conduction band. This difference is yet another indication of significance of real-space electron distributions.

**Figure 2:**
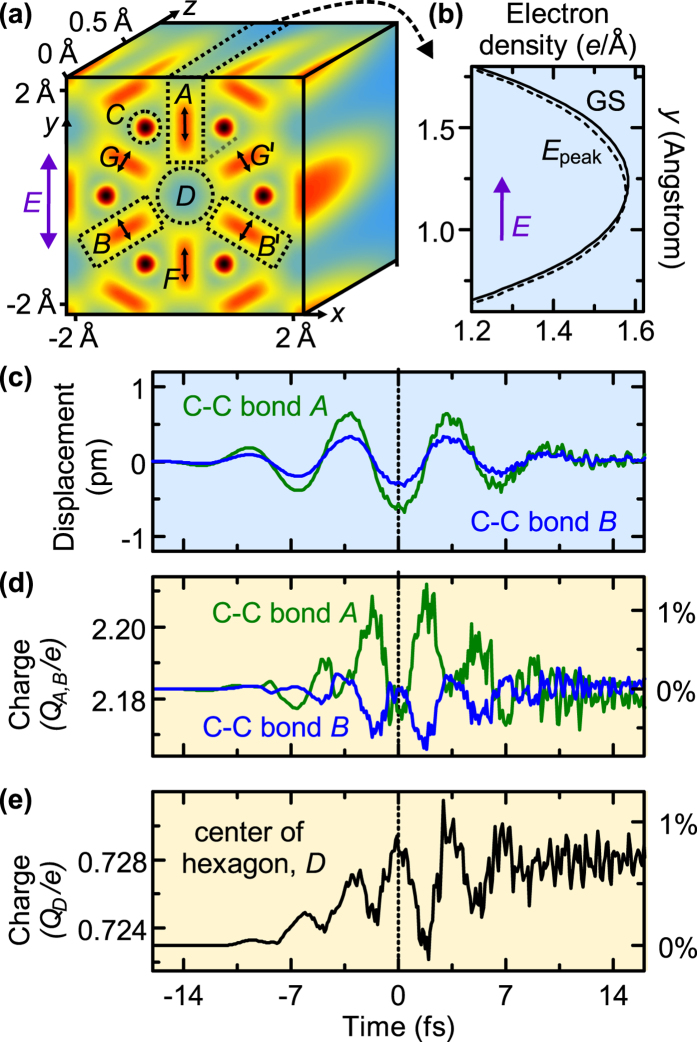
Real-space analysis of sub-cycle laser-induced perturbations of the charge density in graphene. (**a**) Definition of volumes *A*-*D* corresponding to distinct types of electrons. The three facets of the cube display the stationary density of valence electrons in the planes *z* = 0, 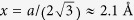, and 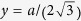; *a* = 2.46 Å is the lattice period. Volumes *A* and *B* cover the bonds between carbon atoms; volumes *C* and *D* cover the nuclei and the hexagon centre, respectively. (**b**) The displacement and deformation of the charge distribution in volume *A* in the ground state (GS, solid) and at the peak of the laser field (dashed) along the direction of the laser polarisation (*y*-axis) and integrated over *x* and *z*. (**c**) Time-dependent displacement of electrons in volumes *A* (green) and *B* (blue); the direction is indicated by the black arrows in panel (**a**). For both bonds, it nearly instantaneously follows the electric field. (**d**) Time dependence of the charge (number of electrons) in volumes *A* (green) and *B* (blue), revealing dynamics at twice the laser frequency. (**e**) Charge in volume *D* around the hexagon centre, increasing stepwise with time as a result of electronic excitations.

To further elucidate the importance of charge movement in real space, we introduce some regions of interest, labelled *A-G* in Fig. 2(a). These regions are chosen to encompass distinguishable areas of electron density. The volumes associated with the C-C sigma bonds form two Bravais sub-lattices, one of which contains regions *A*, *B*, and 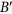, and the other one *F*, *G*, and 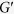. Areas *A* and *B* are 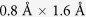, the radius of area *C* is 0.26 Å, and that of area *D* is 0.53 Å. Along the *z* direction, these volumes extend through the entire simulation space. In the stationary state, volumes *A* and *B* contain *Q*_*A*_ = *Q*_*B*_ = 2.2 electrons each, which is close to the expected two electrons per the sp^2^-type sigma bond; volume *C* contains *Q*_*C*_ = 0.29 electrons, and there are *Q*_*D*_ = 0.72 electrons in volume *D*. Together, 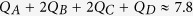 electrons per unit cell, close to the overall number of 8 valence electrons of graphene. This indicates that all relevant charges are covered by our assignment.

In each region, the time-dependent effects of the laser field on the charge density are decomposed into two components: local displacement and redistribution between different volumes. We quantified the displacement by evaluating the first momentum (centre of mass) in each volume. For the C-C bond perpendicular to the laser polarisation (region *A*), Fig. 2(b) compares the ground-state electron density (solid) to that at the instance of maximum field strength (dashed). At this moment, the main effect of the field is displacement of the charge within the bond, though some decrease of the charge is also visible.

Temporal dynamics of displacements and charges are shown in Fig. 2(c–e) for the two types of C-C bonds (volumes *A* and *B*) and for the centre of the graphene hexagon (volume *D*). The field-induced local displacement of the electrons in volumes *A* and *B* is about one picometre; see Fig. 2(c). At all times, the shift is approximately proportional to the laser field. The C-C bond represented by volume *B* also experiences a shift that is proportional to the electric field [Fig. 2(c), green]. The direction of the shift is shown by black arrows in Fig. 2(a) and it is not parallel to the laser field, as one could naively expect. Instead, the centre of mass of volume *B* moves predominantly along the bond. This is due to the polarisability of a bond being larger along its direction than normal to it. The valence charges around the nuclei (volume *C*) shift by only ~0.07 pm in the laser field and give/receive a maximum of only ~0.002 electron. These charges are, therefore, negligible for graphene’s optical and electronic dynamics.

Figure 2(d) shows the time-dependent integrated charge in volumes *A* and *B*. Both traces start at the same amount of charge, ~2.2 electrons, which is close to the charge expected from chemical considerations: two electrons from a sigma bond plus 60°/360° = 1/6 ≈ 0.167 from the one delocalised electron per carbon in the out-of-plane bonds. The charge 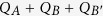 is approximately conserved at all times. Also, in a good approximation, the changes of the charge in the individual regions satisfy the relation 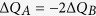. This reflects the symmetry of the system with respect to reflection 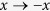 in conjunction with pseudo-spin conservation implying charge conservation within each sub-lattice. As each of the two type-*A* bonds receives its peak charge increase of ~0.03 electrons, each of the four type-*B* bonds loses ~0.015 electrons. Our results show that this charge transfer mainly takes place at 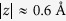, i.e., significantly out of the plane of graphene.

Unlike the charge displacement, the amount of charge in a certain volume is modulated in time with twice the laser frequency, and it is symmetric with respect to the sign of the laser field. Also, the phase of this field-induced charge migration between different types of bonds indicates that the vector potential, rather than the electric field, determines these dynamics: There is little charge transferred at the field extrema, while a maximum amount of charge is transferred between volumes *A* and *B* at the zero electric field. The fact that the magnitude of the electric current is also maximal at the zero field suggests that the atomic-scale charge redistribution shown in Fig. 2(d) is a prerequisite for inducing a significant electric current in undoped graphene. The effective charge transfer between regions *A* and 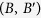 is also largely symmetric with respect to 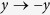 and, consequently, negligibly contributes to net polarisation and current. This charge transfer effect is therefore hidden from far-field spectroscopic characterisation.

Figure 2(e) shows time-dependent charge *Q*_*D*_ in the centre of the unit cell, volume *D*. This charge has local maxima at the crests of the laser field and minima at its zero crossings. On a few-femtosecond time scale, there is gradual accumulation of charge in volume *D* that persists after the pulse end, which is also evident in Fig. 1(e). Physically, this implies that the charge accumulation in the unit cell centre, *D*, has both an adiabatic component (the local maxima following the absolute strength of the laser field) and a non-adiabatic component caused by resonant absorption. This absorption in graphene is broadband^28^, causing rapid dephasing (Landau damping), leading to the stationary residual electronic excitation.

### Experimental feasibility of sub-cycle electron diffraction

Here we propose an experiment for direct visualisation of purely electronic dynamics in graphene, and discuss its feasibility. The following three considerations are critical. First, the spatial resolution must be atomic. Second, temporal resolution must be sub-cycle. Third, there should be sufficient signal-to-noise ratio for the expected charge density changes, which manifest themselves in diffraction as time-dependent changes of Bragg peak intensities. In our case, sub-cycle means a quarter cycle of the laser, i.e. ~1.75 fs for the 2.1-μm driving laser considered here, in order to temporally resolve the largely nonlinear electron motion driven by a strong field, including, e.g., dynamics responsible for third harmonic generation.

Two currently emerging technologies can provide these prerequisites. Free-electron lasers (FELs) can deliver pulses with attosecond substructures^29^,^30^, and significant efforts are being made to scale up the photon energy toward wavelengths that would be sufficiently short for atomic-scale imaging^31^,^32^. The low scattering cross section of X-rays is compensated by the superior average photon flux of FELs; note that a particular peak flux is not required since the experiment is reversible. Some ionisation of the graphene sample eventually caused by a pump or a probe pulse will be rapidly replenished^33^. The second approach is ultrafast electron diffraction^34^,^35^. The de Broglie wavelength of electrons is as small as several picometres for kinetic energy of tens of kiloelectronvolts. The scattering efficiency from graphene is high, ~10^−3^ from the central beam into a typical Bragg spot; see Methods. Ultrashort pulses can be produced by microwave compression^36^,^37^ and, in the case of only one or a few electrons per pulse, simulations predict attosecond pulse durations^38^,^39^. Indeed, there is indirect experimental evidence^40^ of an attosecond electron pulse train^15^.

Here we consider electron diffraction at 30 keV, an energy low enough for efficient scattering and high enough to provide a 7-pm de Broglie wavelength. Diffraction is modelled neglecting inelastic scattering and the electron transit time through the sample, which is a few attoseconds; see Methods. At a realistic 300 kHz pump-probe repetition rate^41^, about 10^9^ single-electron pulses are incident on the sample within one-hour integration time. With a scattering probability of ~10^−3^, this allows realistically low shot noise in the Bragg peak intensities.

**Figure 3:**
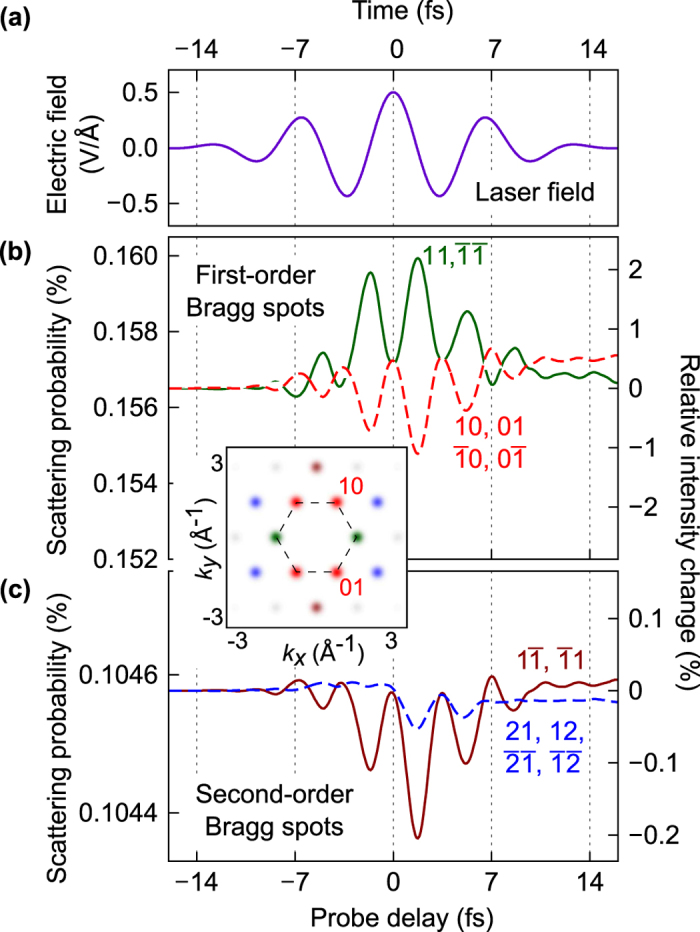
Simulated pump—probe Bragg diffraction. (**a**) Electric field of the laser pulse. (**b**) Scattering probabilities into first-order Bragg spots as functions of the arrival time of a 1-fs electron pulse with a mean electron kinetic energy of 30 keV. The maximum intensity change is 1–2%. (**c**) Scattering probabilities for the second-order Bragg spots. The maximum intensity change is 0.05–0.2%. The inset schematically shows the first Brillouin zone and the positions of the Bragg spots, which are coloured in the same way as the curves in panels (**b**,**c**).

The inset in Fig. 3 shows the expected Bragg pattern, where we have indicated Miller indices for the two primary reciprocal-space vectors. For the first two diffraction orders, Fig. 3(b,c) show the scattering probabilities as functions of the delay between the laser field [Fig. 3(a)] and a 1-fs electron pulse. Notably, the atomic-scale electron dynamics modulate the scattering probabilities by up to 2% within the first diffraction order and up to 0.2% within the second one. Such values are sufficiently high to be experimentally accessible with state-of-the-art pump-probe electron diffraction^42^,^43^.

The time-dependent scattering probabilities shown in Fig. 3 have symmetry that is specific for our experimental geometry: 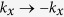 or 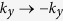, in addition to the general Friedel symmetry of Bragg diffraction. An important observation can be made by comparing Figs 2(d) and 3(b): For Bragg peaks 11 and 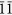, the delay dependence of scattering probabilities looks remarkably similar to the time dependence of the charge in volume *A*, while the charge in volume *B* correlates with the intensities of the other first-order Bragg peaks. This similarity encompasses both the modulation period (half of a laser cycle) and the changes that remain after the interaction. This suggests that significant aspects of the real-space light-driven electronic motion can be reconstructed from time-dependent diffraction experiments.

### Retrieval of time-dependent charge-density maps

Which details can be retrieved? Bragg-peak intensities represent the magnitudes of complex scattering amplitudes, whose phases are not measured. Many advanced techniques exist to circumvent this problem using prior information, but these techniques may require some adaptation to reveal spatially delocalised changes in electron density, which are in contrast to localised atomic-type scatterers in crystallography.

Therefore, we refrain here from algorithmic procedures and report on a reconstruction specific to the symmetry in our example. First, we assume that the initial microscopic scattering potential is known, for example from ground-state calculations or state-of-the-art crystallography^44^,^45^. Second, we assume that the scattering amplitudes maintain their initial phases during the interaction with the laser pulse; see Methods. In this way, we reconstruct the part of the time-dependent electronic motion that is symmetric with respect to the reflection 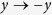, which is hidden from spectroscopy in dipole approximation. We used only the lowest two orders of Bragg scattering as depicted in the inset in Fig. 3.

**Figure 4:**
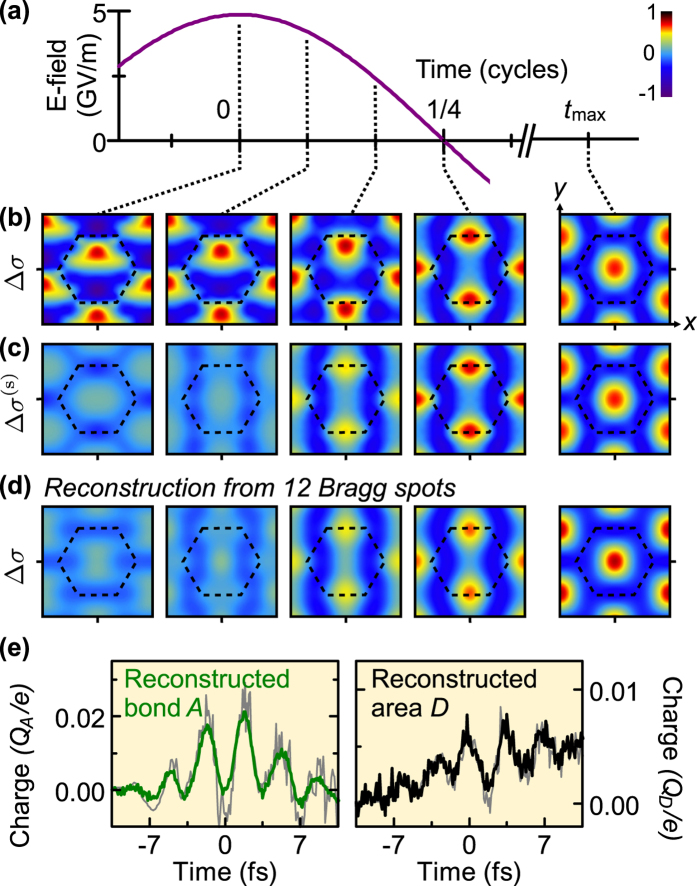
Reconstruction of the atomic-scale electron dynamics from pump-probe diffraction. (**a**) Central laser cycle (violet) and five times of reconstruction. (**b**) Snapshots of the *z*-integrated electron-density change at the chosen moments of time. (**c**) Electron density symmetrised with respect to the reflection 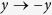. (**d**) Reconstruction of electron density snapshots from the 12 Bragg spots of Fig. 3 assuming no phase change of the ground-state scattering amplitudes. Whenever the charge distribution is symmetric with respect to the reflection 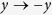, the reconstruction of the real electron density (**b**) is almost perfect. At other times, the symmetric part (**c**) is well reconstructed. (**e**) Time-dependent reconstructed charge in volumes *A* between carbon atoms (green) and volume *D* around the hexagon centre (black) in comparison to the actual simulated results (grey). These results confirm the ability of ultrafast diffraction to recover the atomic-scale, real-space origin of macroscopic optical and electronic phenomena.

Figure 4 shows the results. In Fig. 4(a), we depict four representative times in the laser cycle and, additionally, a time 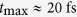, after the pulse end. The electron densities are integrated over *z* because *z*-dependent information is absent in diffraction geometry at normal incidence. Figure 4(b) shows changes in the simulated electron density, Δ*σ*, and Fig. 4(c) shows the Δ*σ*^(s)^ that is symmetric with respect to reflection 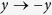, i.e. the part hidden from far-field spectroscopy. The outcomes of the reconstruction are shown in Fig. 4(d). The reconstructed charge-density maps are nearly identical to the *y*-symmetric parts of the actual electronic motion. Figure 4(e) shows the time-dependent charges in regions *A* and *D*, which are reliably reconstructed. A similar quality of reconstruction is also achieved for regions *B* and *C* (not shown). The spatial resolution can be further improved by incorporating more Bragg peaks in the reconstruction, provided that the signal-to-noise ratio is sufficiently large.

### General value of the real-space approach

We conclude by returning to our initial conjecture on the importance of time-dependent real-space recording of electronic motion. A periodic solid can be fully treated in either reciprocal or real space. However, the simplicity of the description or the feasibility of an experiment can define a preferred perspective. In graphene, our real-space analysis has revealed that electrons in different spatial regions respond to an external field in distinctly different ways. In particular, current flows in stripes above and below the plane of the chemical bonds, absorption populates the hexagon centre, the electrons localised at the bonds are periodically displaced along their structural extensions, and charges are periodically transferred in and out of the conducting stripes. These spatial peculiarities of the light-induced electron dynamics suggest the real-space perspective to be a worthwhile concept exposing features that are not easily discernible otherwise.

Real-space measurements will probably contribute most when applied to problems where spectroscopy does not provide sufficient information. They may give new insights into electronic origins of light-induced structural deformations, for example in graphite^46^, vanadium dioxide^15^, charge-density-wave materials^47^ or nanoparticles^42^. Knowing the mechanisms of charge transfer on an atomic scale may be indispensable for understanding strongly-correlated systems, i.e. materials where electron-electron interactions play a major role, as well as the build-up of electron-boson interaction, which has recently been shown to be an ultrafast process in high-temperature superconductors^48^. Identifying structure-function relations for optical phenomena can facilitate design of novel materials and ultrafast electronic devices. Ultrafast electron and X-ray diffraction bear a promise to become important techniques to study light-matter interaction in condensed matter.

## Methods

### The model

We designed a numerical model adapted for studying ultrafast electron diffraction on an atomically thin sample exposed to a strong field. We solve the time-dependent Schrödinger equation (TDSE) in three spatial dimensions for an ensemble of electrons moving in a local “effective” lattice potential *V*(***r***) that is periodic in any plane parallel to the atomic plane: *V*(***r*** + ***a***_1,2_) = *V*(***r***). Here, ***a***_1_ and ***a***_2_ are the lattice unit vectors, both of which are orthogonal to ***e***_*z*_. Due to this periodicity, the Bloch theorem applies. We introduce the in-plane crystal momentum 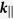, which is a conserved quantity in the velocity gauge. Henceforth we use atomic units and write the TDSE as


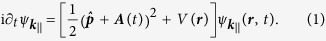


Here, ***A***(*t*) is related to the external electric field by 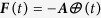. Equation (1) implies the dipole approximation; thus, we neglect the effect of the magnetic field.

Let 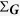 denote a sum over vectors of the reciprocal lattice. For each 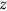, we decompose both the wave function 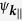 and the latice potential in Fourier series:


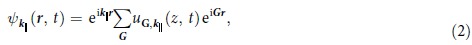



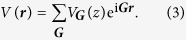


For a function *f*(***r***) that has the periodicity of the lattice, the decomposition coefficients are


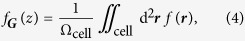


where the integration is performed over the unit-cell area 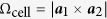. Ansatz (2) translates the TDSE (1) into


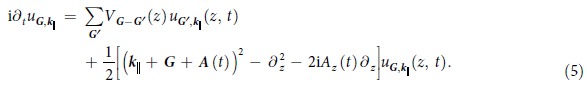


This equation is solved for a set of initial conditions, each of which is defined by 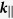 and an initial valence band *n*.

One of the most important physical observables for electron diffraction is the electron density 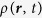, which we decompose into plane waves as well: 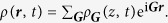 Equations (2) and (4) yield


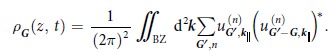


The integral here is taken over the first Brillouin zone (BZ), and the contributions from all initial bands are added together; 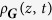 is guaranteed to be non-negative.

**Figure 5:**
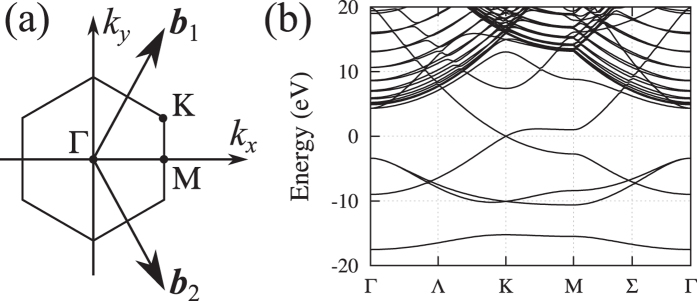
(**a**) The Brillouin zone of graphene and the reciprocal-space vectors. (**b**) The band structure evaluated with our model (the inner-shell bands are not shown). The Fermi energy is *E*_F_ = 0. The energy gaps in the continuum spectrum are due to a relatively small size of the simulation box (51.2 at. u. along the *z*-axis), and they have no impact on electron diffraction.

Our phenomenological lattice potential *V*(***r***) is evaluated as a sum of atomic potentials


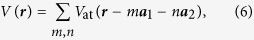


each of which is parametrised as 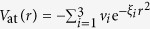 with *v*_1_ = 27.05, *v*_2_ = −10.18, *v*_3_ = 2.08, 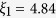, 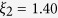, and 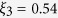. The band structure for this potential is shown in Fig. 5; it is similar to that obtained in *ab initio* simulations^49^. The Fermi velocity in our model is equal to 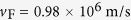 in the direction orthogonal to Γ-K, which is close to the literature value: 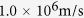
50.

We note that, unlike most pseudopotential methods, our lattice potential supports core states, which is important for electron diffraction. However, to reduce the required number of plane waves in expansion (2), we allow the 1*s* states to be less tightly bound as they are in a carbon atom: the 1*s* state in the potential *V*_at_(*r*) has an energy of −121 eV, while the K-edge of carbon is at 282 eV. This allows us to consider only the reciprocal-space vectors with 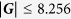. While the presence of the 1*s* state in the atomic potential is essential for obtaining correct nodal structures in the 2*s* and 2*p* states, dynamics of inner-shell electrons in our calculations have a negligible effect on Bragg peaks. Therefore, solving the TDSE, we consider only those initial states that correspond to valence bands.

The laser pulse in our simulations has a central wavelength of 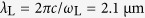, a full width at half maximum (FWHM) duration of 10 fs, and a peak field of 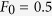 V/Å, which corresponds to a peak intensity of 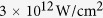. We define the pulse by its vector potential: 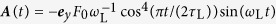 for 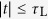 and 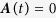 for 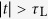.

For graphene, 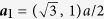, 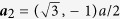, *a* = 4.65 atomic units. Discretising the crystal momentum, we use a Monkhorst grid^51^ with 32 × 32 = 1024 nodes. An 8-th order scheme allows us to evaluate 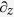 and 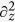 on a relatively coarse *z*-grid: 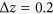. The grid contains 256 nodes, and we apply periodic boundary conditions. For solving the TDSE, we use the 4-th order Magnus propagator^52^,^53^, evaluate matrix exponentials using Expokit^54^, and perform the required sparse-matrix operations with the aid of CXSparse^55^.

### Electron diffraction

We are interested in the intensities of Bragg peaks; their positions do not change as long as the lattice motion can be neglected because the interaction with a laser field preserves lattice periodicity. The first step in modelling elastic scattering of energetic electrons is the evaluation of the scalar potential induced by atomic nuclei, as well as core- and valence-band (VB) electrons. The spatial Fourier transform of the total potential is given by


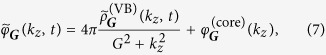


where 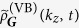 is the Fourier transform of 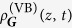 with respect to *z*, and 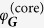 is the ionic potential screened by core electrons; for 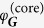, we use the Goedecker-Hartwigsen-Hutter-Teter pseudopotential of carbon^56^. We assume that atomic positions in the lattice have a root-mean-square uncertainty of 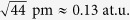 resulting from disorder and thermal motion^57^. We account for this uncertainty by convolving 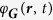 with a Gaussian function.

Two factors simplify the evaluation of scattering probabilities: First, an energetic (tens of keV) projectile electron crosses a graphene sheet within just a few attoseconds and, during this time, the scattering potential can be considered constant. Second, integration over a range of angles and energies at a particular Bragg peak suppresses the interference of waves scattered at different moments of time. We also neglect inelastic transitions that give rise to interference effects discussed in^19^,^58^,^59^. In Supplementary Information, we derive, in the first Born approximation, the following expression for the probability of elastic scattering into a certain Bragg peak:


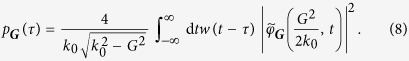


Here, *w*(*t*) is the probability density for a projectile electron to cross the plane *z* = 0 at a time *t*, *k*_0_ is the average momentum of projectile electrons, and *τ* is the delay between the laser and electron pulses. For simulations, we use a kinetic energy of 30 keV 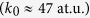 and a Gaussian electron wave packet: 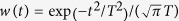 with a FWHM of 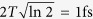.

### Reconstruction of electron density

We add a realistic amount of Poisson-distributed shot noise to the scattering probabilities (8) prior to reconstruction. We model the noise assuming that 10^9^ electrons are available to record Bragg-peak intensities for a given delay between the electron and laser pulses. After that, we account for the symmetry of the considered problem by averaging electron counts in Bragg peaks that are symmetric with respect to the reflection off axes *k*_*x*_ or *k*_*y*_. This ensures both Friedel’s law and the reflection symmetry in the reconstructed electron density.

For reconstruction, we only use the lowest two diffraction orders because, for higher orders, the shot noise dominates the useful signal. For low diffraction orders, the change of *p*_*z*_ upon scattering is negligible: 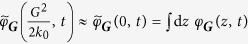 in Eq. (8). In this approximation, the diffraction pattern is determined by the electron density integrated over the direction normal to the sample:


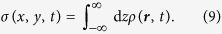


Our reconstruction procedure, inspired by^60^, assumes time-independent phases of scattering amplitudes. In the stationary state of graphene, these amplitudes are real numbers, which reduces the information about their phases to knowing the sign of 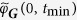. During the laser pulse, 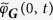 are complex numbers, but the sum of each pair 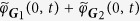 is real if 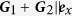. Replacing each such pair of Fourier components with their averaged sum is equivalent to neglecting the imaginary parts of both 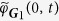 and 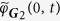, and it is also equivalent to symmetrising the charge density:


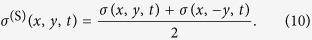


Thus, by assuming constant scattering phases, we reconstruct the charge density symmetrised along the *y*-axis and integrated over *z*.

### Details on the figures

Figures 1(b) and 2b display the sum of electron densities evaluated from all the valence bands:


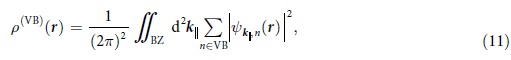


where 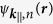 are stationary states. Figure 1(c) illustrates how 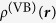 would change if all valence-band electrons within a 2-eV energy range under the Fermi level were excited into the lowest conduction band preserving 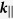. The average current density in Fig. 1(d) is evaluated as


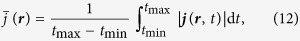


where the time interval between 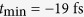 and 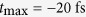 encompasses the laser pulse. The microscopic current density is evaluated using the following equations:


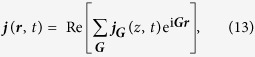



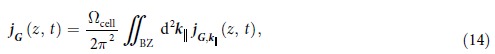



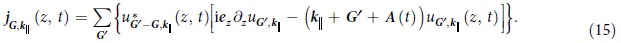


The fast oscillations in Fig. 2(c–e) are due to the coherent superpositions of conduction- and valence-band states. They have a broad frequency spectrum, and the period of these quantum beats is much smaller than the duration of the electron pulse. Therefore, they are not time-resolved in the pump-probe measurement (Fig. 3). In Fig. 3(b,c), we plotted 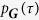 given by Eq. (8).

The symmetrised density in Fig. 4(c) is defined as


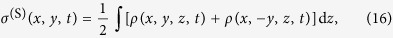


which is the same as Eq. (10). The results shown in Fig. 4(d,e) are reconstructed from data obtained by adding shot noise to the scattering probabilities displayed in Fig. 3, see above.

### Experimental damage considerations

The deposited energy per pulse at a field of 0.5 V/Å is approximately 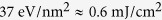. The spot size of the pump laser is determined by the size of the few-femtosecond electron beam, realistically ~50 μm diameter at the sample^61^. Each pump pulse heats up graphene by < 200 K, which involves electron-phonon and phonon-phonon relaxation processes that take their course within picoseconds^62^. At 300 kHz pump-probe repetition rate, about 3.5 mW of average power is absorbed. This is sufficiently low to be removed between pump pulses by heat conduction with a finely spaced TEM mesh^41^. Multiple scattering effects in electron diffraction are negligible because only tiny changes of Bragg intensities are recorded.

## Additional Information

**How to cite this article**: Yakovlev, V. S. *et al.* Atomic-scale diffractive imaging of sub-cycle electron dynamics in condensed matter. *Sci. Rep.* **5**, 14581; doi: 10.1038/srep14581 (2015).

## Supplementary Material

Supplementary Information
