# Supplementary material for: Atomic-scale diffractive imaging of sub-cycle electron dynamics in condensed matter
Source: Sci Rep. 2015 Sep 28;5:14581. doi: 10.1038/srep14581 (PMC4585944; doi:10.1038/srep14581)
Supplement: Supplementary Information [file srep14581-s1.pdf]

Scattering of an energetic electron wave packet by an atomically thin sample

Here, we present a derivation of Eq. (A.8) and discuss the involved approximations.

General considerations

We consider scattering of an electron wave packet from an atomically thin sample (e.g. graphene) placed at $z=0$. Considering the Coulomb interaction between the projectile electron and the sample as a perturbation described by an operator $\hat{V}$, we write the time-dependent Schrödinger equation in the integral form using atomic units:

$$\hat{U}(t_\mathrm{f},t_\mathrm{i}) = \hat{U}_0(t_\mathrm{f},t_\mathrm{i}) - \mathrm{i}\int_{t_\mathrm{i}}^{t_\mathrm{f}} \mathrm{d}t\, \hat{U}(t_\mathrm{f},t)\hat{V}\hat{U}_0(t,t_\mathrm{i}). \tag{1}$$

Here, $\hat{U}_0(t,t_\mathrm{i})$ is the evolution operator without the perturbation, and $\hat{U}(t,t_\mathrm{i})$ is the exact propagator. These operators act on many-electron wave functions that depend on the electrons of the sample, as well as on the projectile electron:

$$|\Psi(t)\rangle = |\psi_\mathrm{s}(t)\rangle \otimes |\psi_\mathrm{p}(t)\rangle. \tag{2}$$

The probability amplitude to make a transition from an initial state $|i\rangle$ to a final state $|f\rangle$, as time goes from t_i to t_f, is given by

$$\langle f|\hat{U}(t_\mathrm{f},t_\mathrm{i})|i\rangle = \langle f|\hat{U}_0(t_\mathrm{f},t_\mathrm{i})|i\rangle - \mathrm{i}\int_{t_\mathrm{i}}^{t_\mathrm{f}} \mathrm{d}t\, \langle f|\hat{U}(t_\mathrm{f},t)\hat{V}\hat{U}_0(t,t_\mathrm{i})|i\rangle. \tag{3}$$

The choice of t_i and t_f is arbitrary, as long as t_i precedes the interaction of the incident wave packet with the sample, and the interaction is accomplished by the moment t_f. Our ultimate goal is to evaluate the probability $p_{\boldsymbol{G}}$ of detecting an electron within a Bragg peak labelled by the reciprocal-space vector $\boldsymbol{G}$.

Without the interaction between the sample and the projectile, their wave functions evolve independently:

$$\hat{U}_0(t,t_\mathrm{i})|i\rangle = |\psi_\mathrm{s}(t)\rangle \otimes \left|\psi_\mathrm{p}^{(\mathrm{i})}(t)\right\rangle, \tag{4}$$

where $|\psi_\mathrm{s}(t)\rangle$ is assumed to be known, and the incident wave packet propagates according to

$$\left|\psi_\mathrm{p}^{(\mathrm{i})}(t)\right\rangle = \int \mathrm{d}^3k\, \mathrm{e}^{-\mathrm{i}\frac{k^2}{2}(t-t_\mathrm{i})} |\boldsymbol{k}\rangle \left\langle \boldsymbol{k}|\psi_\mathrm{p}^{(\mathrm{i})}(t_\mathrm{i})\right\rangle. \tag{5}$$

Here, we have neglected the acceleration of the projectile electron by the laser pulse and introduced plane waves:

$$\langle \boldsymbol{r}|\boldsymbol{k}\rangle = \frac{\mathrm{e}^{\mathrm{i}\boldsymbol{kr}}}{(2\pi)^{3/2}}. \tag{6}$$

Now we make two approximations: we neglect inelastic scattering and apply first-order perturbation theory. The former approximation allows us two use the known $|\psi_\mathrm{s}(t_\mathrm{f})\rangle$ in the final state; that is, we assume that the projectile electron has no effect on the sample. In this case, a possible final state is

$$|f\rangle = |\psi_\mathrm{s}(t_\mathrm{f})\rangle \otimes |\boldsymbol{k}_\mathrm{f}\rangle. \tag{7}$$

The application of perturbation theory consists in replacing $\hat{U}(t_\mathrm{f},t)$ with $\hat{U}_0(t_\mathrm{f},t)$ in the right-hand side of Eq. (3). The interaction between the sample and the projectile being neglected,

$$\langle f|\hat{U}_0(t_\mathrm{f},t) = \mathrm{e}^{-\mathrm{i}\frac{k_\mathrm{f}^2}{2}(t_\mathrm{f}-t)} \langle \boldsymbol{k}_\mathrm{f}| \otimes \langle \psi_\mathrm{s}(t)|, \tag{8}$$

which allows us to re-write Eq. (3) as

$$\langle f|\hat{U}(t_\mathrm{f},t_\mathrm{i})|i\rangle \approx \left\langle \boldsymbol{k}_\mathrm{f}|\psi_\mathrm{p}^{(\mathrm{i})}(t_\mathrm{f})\right\rangle \langle \psi_\mathrm{s}(t_\mathrm{f})|\psi_\mathrm{s}(t_\mathrm{f})\rangle - \mathrm{i}\int_{t_\mathrm{i}}^{t_\mathrm{f}} \mathrm{d}t\, \mathrm{e}^{-\mathrm{i}\frac{k_\mathrm{f}^2}{2}(t_\mathrm{f}-t)} \langle \boldsymbol{k}_\mathrm{f}| \otimes \langle \psi_\mathrm{s}(t)|\hat{V}|\psi_\mathrm{s}(t)\rangle \otimes \left|\psi_\mathrm{p}^{(\mathrm{i})}(t)\right\rangle. \tag{9}$$

The first term on the right-hand side of this equation is irrelevant to us as it describes propagation without scattering, so we drop it. We will also drop the inessential phase factor $\exp[-\mathrm{i}k_\mathrm{f}^2 t_\mathrm{f}/2]$.

Evaluating the matrix element $\langle \boldsymbol{k}_\mathrm{f}| \otimes \langle \psi_\mathrm{s}(t)|\hat{V}|\psi_\mathrm{s}(t)\rangle \otimes \left|\psi_\mathrm{p}^{(\mathrm{i})}(t)\right\rangle$, one can begin by integrating over all the electrons of the sample, which is possible as long as the projectile electron is not entangled with $|\psi_\mathrm{s}(t)\rangle$ at any time. Once this integration

is performed, $\langle \psi_s(t) | \hat{V} | \psi_s(t) \rangle$ can be regarded as a time-dependent single-electron scattering potential, which we denote by $\hat{V}_s(t)$. If, in addition, one neglects the exchange interaction between the projectile and the electrons of the sample, $\hat{V}_s(t)$ becomes a local potential represented by a spatially periodic function, which is the potential induced by the charge density of the sample $\rho(\boldsymbol{r},t)$:

$$V_s(\boldsymbol{r},t) = V_s(\boldsymbol{r}+\boldsymbol{a}_{1,2},t), \tag{10}$$

$$V_s(\boldsymbol{r},t) = -\varphi^{(\mathrm{VB})}(\boldsymbol{r},t) - \varphi^{(\mathrm{core})}(\boldsymbol{r},t), \tag{11}$$

$$\nabla^2\varphi(\boldsymbol{r},t) = -4\pi\rho(\boldsymbol{r},t), \tag{12}$$

where φ denotes the scalar potential, and "VB" stands for valence bands. Exchange interaction would add non-local terms to $\hat{V}_s(t)$, and we assume that projectile electrons are sufficiently energetic to neglect these terms.[1]

Finally, the probability amplitude of detecting a scattered electron with a momentum $\boldsymbol{k}_f$ is given by the following well-known expression:

$$\alpha(\boldsymbol{k}_f) = -\mathrm{i}\int_{t_i}^{t_f} \mathrm{d}t\, \mathrm{e}^{\mathrm{i}\frac{k_f^2}{2}t} \left\langle \boldsymbol{k}_f \middle| \hat{V}_s(t) \middle| \psi_p^{(i)}(t) \right\rangle. \tag{13}$$

Probably the most essential approximation that we have made so far is that of neglecting inelastic transitions, and some comments here are due. The most important argument supporting this approximation is that we are interested in the probability of scattering an electron within a rather small solid angle associated with a certain Bragg spot. Inelastic processes are expected to contribute little to these probabilities because they scatter electrons into a much larger solid angle, and also because the probability of inelastic scattering rapidly decreases with the scattering angle.

We must also mention that inelastic transitions can play an important role when energetic electrons or X-ray pulses are scattered from atoms prepared in a coherent superposition of its eigenstates.[1–5] Here, interference between different quantum pathways plays an essential role. For example, if an atom is in a superposition of eigenstates $|1\rangle$ and $|2\rangle$, then elastic scattering from state $|2\rangle$ can interfere with the quantum path where scattering from state $|1\rangle$ is accompanied by a transition from $|1\rangle$ to $|2\rangle$, provided that the probe pulse is sufficiently broadband, so that the two pathways can lead to the same final energy of the scattered particle (electron or photon). Even if the probability of the latter pathway is much smaller than that of the elastic one, their interference can lead to significant effects, especially in the angular distribution of scattering probabilities. While we cannot strictly rule out the importance of such interference effects in the case of condensed matter, we neglect them for the same reasons why we neglect inelastic scattering in general—to be observable, inelastic scattering must be significant at diffraction angles satisfying the Bragg condition. Also, we consider an electron pulse that is longer than a typical period of quantum oscillations formed by coherent superpositions of conduction- and valence-band states. That is, the fast oscillations present in Figs. 2(c-e), which are particularly visible at the trailing edge of the laser pulse, are not resolved in the Bragg-peak intensities presented in Fig. 3 of the main text.

Probability of Bragg scattering

During one femtosecond, an electron that has a kinetic energy of 10 keV propagates over a distance of $\sim 10^3$ atomic units. Thus, for an atomically thin sample (thickness ~ 1 at. u.), the following approximation can be made:

$$V_s(\boldsymbol{r},t)\psi_i(\boldsymbol{r},t) \approx V_s(\boldsymbol{r},t)\psi_i(x,y,z=0,t)\mathrm{e}^{\mathrm{i}k_z(t)z}, \tag{14}$$

where $k_z(t)$ is defined as

$$k_z(t) = \left(\frac{\partial}{\partial z}\arg\left[\psi_i(\boldsymbol{r},t)\right]\right)\Big|_{z=0}. \tag{15}$$

Furthermore, it is convenient to factor out the time dependence of the incident wave packet:

$$\psi_i(x,y,z=0,t) = \phi(x,y,t)\mathrm{e}^{-\mathrm{i}\varepsilon_0 t}, \tag{16}$$

where

$$\varepsilon_0 = \frac{k_0^2}{2} \tag{17}$$

is the mean projectile energy. Using Eqs. (16), (14), and (6), we rewrite Eq. (13) as

$$\alpha(\boldsymbol{k}_f) = -\frac{\mathrm{i}}{(2\pi)^{3/2}}\int_{t_i}^{t_f}\mathrm{d}t\,\mathrm{e}^{\mathrm{i}\left(\frac{k_f^2}{2}-\varepsilon_0\right)t}\int \mathrm{d}^3r\,\mathrm{e}^{-\mathrm{i}\boldsymbol{k}_f\boldsymbol{r}}V_s(\boldsymbol{r},t)\phi(x,y,t)\mathrm{e}^{\mathrm{i}k_z(t)z}. \tag{18}$$

To proceed, we consider an auxiliary problem of integrating $\exp[-\mathrm{i}\boldsymbol{q}\boldsymbol{r}_\parallel]f(\boldsymbol{r}_\parallel)$ with $\boldsymbol{r}_\parallel=(x,y)$ for a spatially periodic function $f(x,y)$ over a finite number of two-dimensional unit cells:

$$I=\sum_{m=-N}^{N}\sum_{n=-N}^{N}\int_{\text{cell}}\mathrm{d}^2r_\parallel\left(\left.\mathrm{e}^{-\mathrm{i}\boldsymbol{q}\boldsymbol{r}_\parallel}f(\boldsymbol{r}_\parallel)\right|_{\boldsymbol{r}_\parallel\to\boldsymbol{r}_\parallel-m\boldsymbol{a}_1-n\boldsymbol{a}_2}\right).$$

For $f(\boldsymbol{r}_\parallel-\boldsymbol{a}_{1,2})=f(\boldsymbol{r}_\parallel)$,

$$I=\sum_{m=-N}^{N}\sum_{n=-N}^{N}\int_{\text{cell}}\mathrm{d}^2r_\parallel\,\mathrm{e}^{-\mathrm{i}\boldsymbol{q}(\boldsymbol{r}_\parallel-m\boldsymbol{a}_1-n\boldsymbol{a}_2)}f(\boldsymbol{r}_\parallel)=\sum_{m=-N}^{N}\sum_{n=-N}^{N}\mathrm{e}^{\mathrm{i}\boldsymbol{q}(m\boldsymbol{a}_1+n\boldsymbol{a}_2)}\int_{\text{cell}}\mathrm{d}^2r_\parallel\,\mathrm{e}^{-\mathrm{i}\boldsymbol{q}\boldsymbol{r}_\parallel}f(\boldsymbol{r}_\parallel)=$$

$$=\frac{\sin\left(\boldsymbol{q}\boldsymbol{a}_1\left(N+\frac{1}{2}\right)\right)\sin\left(\boldsymbol{q}\boldsymbol{a}_2\left(N+\frac{1}{2}\right)\right)}{\sin\left(\frac{1}{2}\boldsymbol{q}\boldsymbol{a}_1\right)\sin\left(\frac{1}{2}\boldsymbol{q}\boldsymbol{a}_2\right)}\int_{\text{cell}}\mathrm{d}^2r_\parallel\,\mathrm{e}^{-\mathrm{i}\boldsymbol{q}\boldsymbol{r}_\parallel}f(\boldsymbol{r}_\parallel).$$

The auxiliary problem that we are solving here is that of a spatially constrained electron beam, where $\phi(x,y,t)$ is only nonzero if there are integer numbers $|m|\le N$ and $|n|\le N$ such that $\boldsymbol{r}_\parallel-m\boldsymbol{a}_1-n\boldsymbol{a}_2$ lies within the central primitive unit cell. Later, we will take the limit $N\to\infty$. For simplicity, we consider the case of normal incidence, which allows us to substitute $\phi(x,y,t)$ with $\phi(t)$. Also, we normalise the incident wave packet to have one electron per unit cell:

$$\int_{-\infty}^{\infty}\mathrm{d}z\int_{\text{cell}}\mathrm{d}r_\parallel^2\left|\psi_{\mathrm{p}}^{(\mathrm{i})}(\boldsymbol{r},t)\right|^2\equiv 1, \tag{19}$$

so that there are N^2 electrons in the wave packet (we use this normalisation just for convenience only, and it does not affect the final expression for scattering probabilities). The probability density for a particular electron to be scattered into a plane wave with a momentum $\boldsymbol{k}_\mathrm{f}$ is given by

$$p(\boldsymbol{k}_\mathrm{f})=|\alpha(\boldsymbol{k}_\mathrm{f})|^2=\frac{S(\boldsymbol{k}_\mathrm{f})}{(2\pi)^3}\left|\int_{t_\mathrm{i}}^{t_\mathrm{f}}\mathrm{d}t\,\mathrm{e}^{\mathrm{i}\left(\frac{k_\mathrm{f}^2}{2}-\varepsilon_0\right)t}\phi(t)\int_{\text{cell}}\mathrm{d}^2r_\parallel\,\mathrm{e}^{-\mathrm{i}\boldsymbol{k}_\mathrm{f}\boldsymbol{r}_\parallel}\int_{-\infty}^{\infty}\mathrm{d}z\,\mathrm{e}^{\mathrm{i}[k_z(t)-\boldsymbol{e}_z\boldsymbol{k}_\mathrm{f}]z}V_\mathrm{s}(\boldsymbol{r},t)\right|^2, \tag{20}$$

where $\boldsymbol{e}_z$ is a unit vector pointing along the z axis, and $S(\boldsymbol{k}_\mathrm{f})$ is the structure factor:

$$S(\boldsymbol{k}_\mathrm{f})=\frac{1}{N^2}\frac{\sin^2\left(\boldsymbol{k}_\mathrm{f}\boldsymbol{a}_1\left(N+\frac{1}{2}\right)\right)\sin^2\left(\boldsymbol{k}_\mathrm{f}\boldsymbol{a}_2\left(N+\frac{1}{2}\right)\right)}{\sin^2\left(\frac{1}{2}\boldsymbol{k}_\mathrm{f}\boldsymbol{a}_1\right)\sin^2\left(\frac{1}{2}\boldsymbol{k}_\mathrm{f}\boldsymbol{a}_2\right)}. \tag{21}$$

Note that that $S(\boldsymbol{k}_\mathrm{f})$ only depends on the projection of $\boldsymbol{k}_\mathrm{f}$ onto the (k_x,k_y) plane.

As N increases, $S(\boldsymbol{k}_\mathrm{f})$ becomes a function sharply peaked at the positions of Bragg spots: $\boldsymbol{G}=n_1\boldsymbol{b}_1+n_2\boldsymbol{b}_2$, where $n_{1,2}\in\mathbb{Z}$, and the reciprocal-space vectors are defined by

$$\boldsymbol{a}_i\boldsymbol{b}_j=2\pi\delta_{ij}. \tag{22}$$

Indeed, $\sin\left[(n_1\boldsymbol{b}_1+n_2\boldsymbol{b}_2)\boldsymbol{a}_i/2\right]=\sin\left[\pi\left(n_1\delta_{1,i}+n_2\delta_{2,i}\right)\right]=0$. In the limit $N\to\infty$, the integration of $S(\boldsymbol{q})$ over a particular Bragg spot can be performed analytically using

$$\lim_{N\to\infty}\int_{-\pi}^{\pi}\frac{\sin^2\left(\left(N+\frac{1}{2}\right)x\right)}{N\sin^2\left(\frac{x}{2}\right)}\,\mathrm{d}x=4\pi. \tag{23}$$

The result is independent of $\boldsymbol{G}$:

$$\lim_{N\to\infty}\iint_{\text{near }\boldsymbol{G}}\mathrm{d}q_x\,\mathrm{d}q_y\,S(\boldsymbol{q})=\Omega_{\text{BZ}}\lim_{N\to\infty}\int_{-1/2}^{1/2}\mathrm{d}s_1\int_{-1/2}^{1/2}\mathrm{d}s_2\,S\big((n_1+s_1)\boldsymbol{b}_1+(n_2+s_2)\boldsymbol{b}_2\big)=$$

$$=\Omega_{\text{BZ}}\lim_{N\to\infty}\left(\int_{-\pi}^{\pi}\mathrm{d}x\,\frac{\sin^2\left(\left(N+\frac{1}{2}\right)x\right)}{2\pi N\sin^2(x/2)}\right)^2=\Omega_{\text{BZ}}\left(\lim_{N\to\infty}\int_{-\pi}^{\pi}\mathrm{d}x\,\frac{\sin^2\left(\left(N+\frac{1}{2}\right)x\right)}{2\pi N\sin^2(x/2)}\right)^2=4\Omega_{\text{BZ}}.$$

Here, the size of the Brillouin zone $\Omega_{\text{BZ}}=(2\pi)^2/\Omega_{\text{cell}}$ appeared as a Jacobian as we changed the integration variables.

Thus, the probability for an electron to get scattered into a Bragg spot $\boldsymbol{G}$ is equal to

$$p_{\boldsymbol{G}} = \int_{-\infty}^{\infty} \mathrm{d}k_{\mathrm{f},z} \lim_{N\to\infty} \iint_{\text{near } \boldsymbol{G}} \mathrm{d}k_{\mathrm{f},x}\, \mathrm{d}k_{\mathrm{f},x}\, p(\boldsymbol{k}_{\mathrm{f}}) =$$

$$= \frac{4\Omega_{\mathrm{BZ}}}{(2\pi)^3} \int_{-\infty}^{\infty} \mathrm{d}k_{\mathrm{f},z} \left| \int_{t_{\mathrm{i}}}^{t_{\mathrm{f}}} \mathrm{d}t\, \mathrm{e}^{\mathrm{i}\left(\frac{k_{\mathrm{f},z}^2+G^2}{2}-\varepsilon_0\right)t} \phi(t) \int_{\mathrm{cell}} \mathrm{d}^2 r_{\parallel}\, \mathrm{e}^{-\mathrm{i}\boldsymbol{G}\boldsymbol{r}_{\parallel}} \int_{-\infty}^{\infty} \mathrm{d}z\, \mathrm{e}^{\mathrm{i}\left[k_z(t)-k_{\mathrm{f},z}\right]z} V_{\mathrm{s}}(\boldsymbol{r},t) \right|^2 .$$

We can re-write it more compactly by introducing the spatial Fourier transform of the scalar potential $\varphi(\boldsymbol{r},t) = -V_{\mathrm{s}}(\boldsymbol{r},t)$:

$$\tilde{\varphi}_{\boldsymbol{G}}(k_z,t) = -\frac{1}{\Omega_{\mathrm{cell}}} \int_{\mathrm{cell}} \mathrm{d}^2 r_{\parallel} \int_{-\infty}^{\infty} \mathrm{d}z\, \mathrm{e}^{-\mathrm{i}\boldsymbol{G}\boldsymbol{r}_{\parallel}-\mathrm{i}k_z z} V_{\mathrm{s}}(\boldsymbol{r},t). \tag{24}$$

The result is

$$p_{\boldsymbol{G}} = \frac{2\Omega_{\mathrm{cell}}}{\pi} \int_{-\infty}^{\infty} \mathrm{d}k \left| \int_{-\infty}^{\infty} \mathrm{d}t\, \mathrm{e}^{\mathrm{i}\left(\frac{k^2+G^2}{2}-\varepsilon_0\right)t} \phi(t)\, \tilde{\varphi}_{\boldsymbol{G}}\big(k-k_z(t),t\big) \right|^2, \tag{25}$$

where we have set the initial time $t_{\mathrm{i}} \to -\infty$ and the final time $t_{\mathrm{f}} \to \infty$.

We can further simplify this general result if the Fourier transform of the scattering potential $\tilde{\varphi}_{\boldsymbol{G}}$ varies little within the bandwidth of the incident electron wave packet. More precisely, we make the following approximation:

$$\tilde{\varphi}_{\boldsymbol{G}}\big(k-k_z(t),t\big) \approx \tilde{\varphi}_{\boldsymbol{G}}\left(\pm\sqrt{2\varepsilon_0 - G^2} - k_z(t),t\right). \tag{26}$$

The choice of the sign on the right-hand side of this equation depends on the propagation direction of projectile electrons. For definiteness, we take $\langle k_{\mathrm{f},z}\rangle > 0$ and use $+\sqrt{2\varepsilon_0 - G^2}$. Replacing the integral over k by an integral over

$$\omega = \frac{k^2+G^2}{2} - \varepsilon_0 \tag{27}$$

and noticing that only a small range of k near $\pm(\varepsilon_0 - G^2/2)$ contributes to $p_{\boldsymbol{G}}$, which allows us to integrate over ω from $-\infty$ to ∞, we obtain

$$p_{\boldsymbol{G}} \approx \frac{2\Omega_{\mathrm{cell}}}{\pi} \int_{-\infty}^{\infty} \frac{\mathrm{d}\omega}{\sqrt{2\varepsilon_0 - G^2}} \left| \int_{-\infty}^{\infty} \mathrm{d}t\, \mathrm{e}^{\mathrm{i}\omega t} \phi(t)\, \tilde{\varphi}_{\boldsymbol{G}}\left(\sqrt{\varepsilon_0 - G^2} - k_z(t),t\right) \right|^2 =$$

$$= \frac{4\Omega_{\mathrm{cell}}}{\sqrt{2\varepsilon_0 - G^2}} \int_{-\infty}^{\infty} \mathrm{d}t \left| \phi(t)\, \tilde{\varphi}_{\boldsymbol{G}}\left(\sqrt{2\varepsilon_0 - G^2} - k_z(t),t\right) \right|^2. \tag{28}$$

The last step here has been made using Parseval's theorem. At this point, it is convenient to use the central momentum of the projectile electrons $k_0 = \sqrt{2\varepsilon_0}$ (see Eq. (17)). The number of electrons per unit area crossing the plane $z = 0$ during an infinitesimally small time interval $\mathrm{d}t$ is $k_z(t)|\phi(t)|^2\mathrm{d}t$. Since we have normalised the wave function of the incident wave packet to have one electron per unit cell, the probability density for a particular projectile electron to interact with the sample at a time t is given by

$$w(t) = k_z(t)\Omega_{\mathrm{cell}}|\phi(t)|^2. \tag{29}$$

Expressing $p_{\boldsymbol{G}}$ via k_0 and $w(t)$, we obtain

$$p_{\boldsymbol{G}} \approx \frac{4}{\sqrt{k_0^2 - G^2}} \int_{-\infty}^{\infty} \mathrm{d}t\, \frac{w(t)}{k_z(t)} \left| \tilde{\varphi}_{\boldsymbol{G}}\left(\sqrt{k_0^2 - G^2} - k_z(t),t\right) \right|^2. \tag{30}$$

As the final two approximations, we neglect the time dependence of the projectile momentum at $z = 0$, assuming that the incident wave packet is sufficiently long and its chirp is sufficiently small:

$$k_z(t) \approx k_0, \tag{31}$$

and we assume that the scattering angle is small: $k_0 \gg G$. Therefore, we approximate

$$\sqrt{k_0^2 - G^2} - k_z(t) \approx -\frac{G^2}{2k_0}. \tag{32}$$

As long as $V_\mathrm{s}(\boldsymbol{r},t)$ is a real-valued function, $\tilde{\varphi}_{\boldsymbol{G}}(-k,t) = \tilde{\varphi}^*_{\boldsymbol{G}}(k,t)$. Finally, we obtain the following approximation to the more general result given by Eq. (25):

$$p_{\boldsymbol{G}} \approx \frac{4}{k_0\sqrt{k_0^2 - G^2}} \int_{-\infty}^{\infty} \mathrm{d}t\, w(t) \left| \tilde{\varphi}_{\boldsymbol{G}}\left(\frac{G^2}{2k_0}, t\right)\right|^2. \tag{33}$$

Eq. (A.8) in the Methods section differs from this expression only by the explicitly introduced delay of the probe pulse τ.

References

1. Shao, H.-C. & Starace, A. F. Imaging coherent electronic motion in atoms by ultrafast electron diffraction. *Phys. Rev. A* **88**, 062711 (2013).

2. Dixit, G., Vendrell, O. & Santra, R. Imaging electronic quantum motion with light. *Proc. Natl. Acad. Sci. U.S.A.* **109**, 11636–40 (2012).

3. Dixit, G. & Santra, R. Role of electron-electron interference in ultrafast time-resolved imaging of electronic wavepackets. *J. Chem. Phys.* **138**, 134311 (2013).

4. Shao, H.-C. & Starace, A. F. Imaging electronic motions in atoms by energy-resolved ultrafast electron diffraction. *Phys. Rev. A* **90**, 032710 (2014).

5. Santra, R., Dixit, G. & Slowik, J. M. Comment on “How to observe coherent electron dynamics directly”. *Phys. Rev. Lett.* **113**, 189301 (2014).
